# Telepsychotherapie als Chance und Herausforderung: Eine longitudinale Mixed-Methods Studie

**DOI:** 10.1007/s00729-021-00169-2

**Published:** 2021-04-27

**Authors:** Claudia Höfner, Markus Hochgerner, Gerd Mantl, Robert Stefan, Julia Stammer

**Affiliations:** Österreichischer Arbeitskreis für Gruppentherapie und Gruppendynamik, Lenaugasse 3, 1080 Wien, Österreich

**Keywords:** COVID-19, Internetbasierte Psychotherapie, Mixed-Methods, Psychotherapeutische Beziehung, Psychotherapeutische Technik, Internetbasierte Therapie, Telepsychotherapie, COVID-19, Mixed-methods, Psychotherapeutic relationship, Psychotherapeutic technique, Remote psychotherapy, Telepsychotherapy

## Abstract

Seit Beginn der COVID-19 Krise und der sukzessiven Lockdowns sind Psychotherapeut*innen plötzlich gefordert, auf Telepsychotherapie (TEP) umzustellen. Die vorliegende Studie untersucht, wie dieser Umstieg bewältigt wird und wie erfahren sich Psychotherapeut*innen im Umgang mit TEP fühlen, welche Veränderungen in der therapeutischen Beziehung wahrgenommen werden und welche Behandlungstechniken in der TEP als besonders geeignet erscheinen. Die Untersuchungen wurden longitudinal mit zwei Erhebungszeitpunkten mit einem Mixed-Methods Forschungsdesign durchgeführt. Es zeigt sich, dass der Umstieg auf TEP gut bewältigt wird und die Erfahrung mit speziellen Apps und Videotelefonie signifikant zunimmt. Die Ergebnisse der Untersuchung legen für die vorliegende Stichprobe eine gewisse Adaptierungsfähigkeit der Psychotherapeut*innen nahe und zeigen, dass durch TEP keine allgemeine Tendenz zur Verschlechterung der Qualität der therapeutischen Beziehung wahrgenommen wird und dass mit der Zeit eine Adaptierung der therapeutischen Techniken aus der Face-to-Face Situation für die TEP vollzogen wird.

## Abstract (en)

Since the onset of the COVID-19 crisis and successive lockdowns, psychotherapists are challenged to rapidly transition to telepsychotherapy (TEP). The current study investigates how the sudden change is managed and how experienced psychotherapists feel in dealing with TEP, which changes in therapeutic relationship are perceived, and which treatment techniques appear to be particularly appropriate in TEP. The research was conducted longitudinally with two data collection points using a mixed-methods research design. It is found that the switch to TEP is well managed and that experience with specific apps and video telephony increased significantly. The results of the study suggest a certain adaptability of psychotherapists for the present sample and show that there is no general tendency in perceiving a deterioration in the quality of the therapeutic relationship through TEP and that, over time, therapeutic techniques are being adapted from the face-to-face situation to TEP.

## Einleitung

Am 16. März begann in Österreich der erste COVID-19-Lockdown. In der anfangs unübersichtlichen Situation wurden in der niedergelassenen Gesundheitsversorgung Ordinationen und Praxen weitgehend geschlossen, nach Möglichkeit auf Versorgung via Telekommunikation umgestellt. Arbeit im Home-Office und Neologismen wie Distance-Learning oder Social-Distancing prägen seither den Alltag. Auch die Psychotherapie ging auf Distanz (Markowitz et al. 2020; Swartz 2020).

Erfreulicherweise wurde rasch und unbürokratisch seitens der Gesundheitsbehörde und der Sozialversicherungsträger zu Beginn der Krise die Honorierung von Telepsychotherapie (TEP) via Internet oder Telefon ermöglicht (ÖBVP 2020). Damit ist TEP, welcher viele Psychotherapeut*innen bis dahin mit Skepsis begegneten, plötzlich zur Routinepraxis für laufende und neue Psychotherapien geworden (Swartz 2020). Derzeit wird auch in Österreich engagiert erforscht, wie sich die COVID-19 Krise in der Psychotherapie mit Blick auf TEP auswirkt und wie Psychotherapeut*innen den Übergang und die neue Situation bewältigen (Höfner et. al. 2021, Mantl et. al. 2021, Humer et al. 2020; Korecka et al. 2020; Probst et al. 2020).

Empirische Evidenz zur Wirksamkeit von TEP ist zwar noch begrenzt, die vorhandene Studienlage ist aber vielversprechend (Swartz 2020). Markowitz et al. (2020) konstatieren in einem Review, dass die Forschung zu TEP bis zum Beginn der COVID-19 Krise vielfach mit selektiven Populationen gearbeitet hat (z. B. HIV-positive Patient*innen oder Frauen mit postpartaler Depression in ruralen Gebieten). Das ist nun anders, denn plötzlich sind alle Psychotherapeut*innen mit allen Populationen gefordert, TEP oder zumindest gemischte Psychotherapie („*blended therapy*“, eine Kombination von Face-to-Face und TEP) als aktuelle Routinepraxis anzubieten. Außerdem wird von den Review-Autor*innen zu bedenken gegeben, dass die TEP oftmals als Ergänzung zu herkömmlicher Psychotherapie angewendet wurde und dass überwiegend kognitiv behaviorale Ansätze untersucht wurden, welche hauptsächlich mit Sprachtelefonie arbeiteten. Wenig Daten liegen demnach zu anderen methodischen Ansätzen in der TEP und zu Videotherapie vor. Daraus ergibt sich, dass die Forschungsliteratur vor der COVID-19 Krise in Bezug auf die gegenwärtige Situation nur bedingt aussagekräftig ist; Chancen und Herausforderungen werden nunmehr deutlicher.

TEP leistet etwas anderes und etwas mehr als die herkömmliche Psychotherapie. Zum einen ist da die Gesundheitsversorgung in Zeiten von Lockdowns zu nennen, zum anderen ermöglicht sie Behandlung und Supervision, wenn die Face-to-Face Situation aufgrund großer geographischer Distanzen nicht möglich ist. Wenig überraschend wird in dem Review von Markowitz et al. (2020) häufig die eingeschränkte oder nicht vorhandene Face-to-Face Begegnung erwähnt, welche das für die Psychotherapie so wichtige Wahrnehmen, Verstehen und Ausdrücken von Emotionen und den damit verbundenen nonverbal-gestischen Ausdruck und Austausch zum Abgleich im therapeutischen Kontakt erheblich erschwert.

Es ist auch nicht möglich, Patient*innen bei Bedarf ein Taschentuch anzubieten, um nonverbal Mitgefühl zum Ausdruck zu bringen und Trost zu spenden. In einer Untersuchung von McBeath et al. (2020) kurz nach Beginn der COVID-19 Krise werden Gefühle der Isolation in Sitzungen, Schwierigkeiten beim Aufrechterhalten der therapeutischen Beziehung und mit all dem einher schnelle Ermüdung und ein Gefühl mangelnder Kompetenz und Wirksamkeit genannt. Nichtsdestotrotz gibt die überwiegende Mehrheit der Befragten dieser Studie an, dass TEP wirksam ist und sich die behandelten Personen wohl fühlen würden.

Mit Anhalten der COVID-19 Krise wird deutlich, dass neben Querschnittsuntersuchungen auch Entwicklungen und Veränderungen im Umgang mit TEP longitudinal zu untersuchen sind (Korecka et al. 2020). Der vorliegende Artikel setzt hier an und präsentiert Ergebnisse laufender Untersuchungen zu Veränderungen in der Psychotherapie durch die COVID-19 Krise, in welchen Wahrnehmungen und Reflexionen von Psychotherapeut*innen in Österreich hinsichtlich des Umgangs mit TEP erhoben werden. Die Untersuchungen wurden longitudinal mit mehreren Erhebungszeitpunkten in einem Mixed-Methods Forschungsdesign durchgeführt.

Es wird nachfolgend dargestellt, wie der plötzliche Umstieg bewältigt wurde und wie erfahren sich Psychotherapeut*innen im Umgang mit TEP fühlen, wobei hier keine konkreten Hypothesen zugrunde gelegt wurden. Es wurde untersucht, welche Auswirkungen auf die therapeutische Beziehung wahrgenommen werden, wobei anhand des Reviews von Markowitz et al. (2020) und der Ergebnisse von McBeath et al. (2020) die Hypothese zugrunde lag, dass die Qualität derselben durch TEP eher negativ beeinträchtigt wird. Es wurde untersucht, welche Behandlungstechniken in der TEP als besonders geeignet erscheinen, wobei hier die vom Forschungsteam angenommene Hypothese zugrunde lag, dass etwa die Arbeit mit leib- und körperorientierten Interventionen oder kreativen Medien unter TEP leidet. Von Interesse war bei allen Fragen, wie sich der Umgang mit TEP über die Zeit verändert.

## Studiendesign, Messzeitpunkte und Stichprobe

Die Erhebung erfolgte auf Basis einer Online-Befragung über SociSurvey zu drei Testzeitpunkten (t_1_: 06.04.–30.04.2020, t_2_: 12.05.–14.06.2020, t_3_: 20.11.–19.12.2020) mittels eines Fragebogens^1^, welcher offene und geschlossene Fragen kombiniert. Es wurde an Psychotherapeut*innen des Österreichischen Arbeitskreises für Gruppentherapie und Gruppendynamik (ÖAGG) über E‑Mail ein Link zu einer Online-Befragung geschickt. Der Fragebogen umfasste Fragen zur therapeutischen Beziehung sowie zu den Erfahrungen mit neuen Medien in der Psychotherapie und zu den Einschätzungen der Veränderungen der Themen in der Behandlung. Es wurden des Weiteren auch standardisierte Fragebögen^2^ eingesetzt, welche jedoch im Rahmen einer weiteren Veröffentlichung ausgewertet werden (Mantl et. al. 2021). Da der Messzeitpunkt t_3_ derzeit noch ausgewertet wird, werden nachfolgend ausgewählte Ergebnisse aus t_1_ und t_2_ vorgestellt. Der Fragebogen blieb bei den ersten beiden Messzeitpunkten unverändert.

In Österreich gibt es 23 anerkannte psychotherapeutische Verfahren, wobei diese den vier Grundströmungen psychodynamisch, humanistisch, systemisch und verhaltensorientiert zugeordnet werden können (BMSGPK 2020). Die humanistische Orientierung ist bei allen Messzeitpunkten mit über 69 % überrepräsentiert. Das Alter der Psychotherapeut*innen wurde in 5‑Jahres-Kategorien erfragt. Die Teilnahme an der Befragung war freiwillig und jederzeit ohne Nachteil abzubrechen. Beim ersten Messzeitpunkt t_1_ liegen 175 (79,4 % Frauen) vollständig ausgefüllte Online-Fragebögen vor. Median und Modus der erhobenen Altersangaben liegen bei 7, also der Kategorie 50–54 Jahre. 54 Teilnehmer*innen (30,9 %) befanden sich zum Zeitpunkt der Befragung noch in Ausbildung unter Supervision. Beim zweiten Messzeitpunkt t_2_ liegen 177 (79,1 % Frauen) vollständig ausgefüllte Online-Fragebögen vor. Das Alter lag mit einem Median von 7 bei der Kategorie 50–54 Jahre und mit einem Modus von 8 bei der Kategorie 55–59 Jahre. 57 Teilnehmer*innen (32,2 %) befanden sich zum Zeitpunkt der Befragung noch in Ausbildung unter Supervision.

Da eine vollständige Anonymisierung ohne Möglichkeit von Rückschlüssen auf einzelne Personen zugesichert wurde, kam es nach den Erhebungen zu einer strengen, manuellen Zuordnung der Fälle in Bezug auf bestimmte Kriterien (Geschlecht, Altersgruppe, Bundesland, Bildung, Familienstand, psychotherapeutisches Verfahren) um übereinstimmende Fälle im Sinne der Messwiederholung bzw. statistischen Zwillingen zu identifizieren. Es kam somit nach der paarweisen Zuordnung zu einer Fallzahl von 53 Psychotherapeut*innen (77,4 % Frauen), welche über die ersten beiden Messzeitpunkte hinweg teilnahmen. Das Alter lag mit einem Median von 6 bei der Kategorie 45–49 Jahre und mit einem Modus von 5 bei der Kategorie 40–44 Jahre. 17 Teilnehmer*innen (32,1 %) befanden sich zum Zeitpunkt der Befragung noch in Ausbildung unter Supervision.

## Umstieg auf Telepsychotherapie (TEP)

Die quantitative Auswertung zeigt, dass ein größerer Teil der teilnehmenden Psychotherapeut*innen (t_1_: 65,14 %, t_2_: 63,84 %) nicht im Angestelltenverhältnis tätig ist. Aufgrund der rasch eingeräumten Möglichkeit der Kassenfinanzierung wie bei regulärer Psychotherapie konnten so gut wie alle Psychotherapeut*innen TEP durchführen. Lediglich etwa 14 % der Teilnehmenden in einem Angestelltenverhältnis konnten diese Möglichkeit in der Institution nicht nutzen.

Es zeigt sich, dass 92 % der Befragten zum Zeitpunkt t_1_ und 75,1 % zum Zeitpunkt t_2_ TEP zur Behandlung der Patient*innen verwendeten und dass trotz gelockerter Lockdowns im Mai und Juni 2020 eine große Anzahl von Behandlungen weiterhin mit TEP durchgeführt wurde. Psychotherapeut*innen wurden befragt, wie sehr sie sich der TEP gewachsen fühlen, wobei 1 für sehr wenig und 5 für sehr stark stand. Die Deskriptivstatistik zeigt, dass sich Psychotherapeut*innen bei t_1_ (sehr wenig 1: *N* = 6, 2: *N* = 11, 3: *N* = 51, 4: *N* = 60, sehr stark 5: *N* = 47, *N*_gesamt_ *=* 175) und bei t_2_ (sehr wenig 1: *N* *=* 5, 2: *N* *=* 12, 3: *N* *=* 36, 4: *N* *=* 67, sehr stark 5: *N* *=* 57, *N*_gesamt_ *=* 177) mit einem Median von 4 dieser speziellen therapeutischen Arbeit mittels TEP „stark“ gewachsen fühlen. Es zeigt sich, dass sich Psychotherapeut*innen tendenziell etwas mehr bei t_2_ dieser speziellen therapeutischen Arbeit mittels TEP gewachsen fühlen.

Mit Blick auf die Unterschiede hinsichtlich der Erfahrung mit Sprachtelefonie und Videotelefonie zeigt sich, dass Psychotherapeut*innen sich schon bei t_1_ (völlig unerfahren 1: *N* *=* 0, 2: *N* *=* 3, 3: *N* *=* 21, 4: *N* *=* 47, äußerst erfahren 5: *N* *=* 95, *N*_gesamt_ *=* 175) und tendenziell etwas mehr bei t_2_ (völlig unerfahren 1: *N* *=* 4, 2: *N* *=* 8, 3: *N* *=* 23, 4: *N* *=* 56, äußerst erfahren 5: *N* *=* 95, *N*_gesamt_ *=* 177) – wobei 5 der Höchstwert wäre – „sehr erfahren“ mit Sprachtelefonie fühlen. Der Median liegt zu beiden Messzeitpunkten bei 5, also „sehr erfahren“. Betrachtet man die Messwerte aus der paarweisen Zuordnung, so zeigt sich, dass es keinen statistisch signifikanten Unterschied in der Erfahrung im Kontakt über Sprachtelefonie in Abhängigkeit des Messzeitpunkts gibt (asymptotischer Wilcoxon-Test: z *=* −0,065, *p* *=* 0,948, *n* *=* 53). Deutlich anders ist das Ergebnis bei Videotelefonie, bei der Psychotherapeut*innen sich bei t_1_ (völlig unerfahren 1: *N* *=* 35, 2: *N* *=* 25, 3: *N* *=* 49, 4: *N* *=* 45, äußerst erfahren 5: *N* *=* 21, *N*_gesamt_ *=* 175) und bei t_2_ (völlig unerfahren 1: *N* *=* 17, 2: *N* *=* 27, 3: *N* *=* 48, 4: *N* *=* 59, äußerst erfahren 5: *N* *=* 26, *N*_gesamt_ *=* 177) mit einem Median von 3 bei beiden Messzeitpunkten nur „eher erfahren“ fühlen. Hier ist über den Zeitraum von t_1_ und t_2_ eine geringe Steigerungstendenz erkennbar, welche bei Betrachtung der Messwerte aus der paarweisen Zuordnung einen statistisch signifikanten Unterschied zeigt (asymptotischer Wilcoxon-Test: z *=* −2,631, *p* *=* 0,009, *n* *=* 53). Die Größe des statistischen Effekts liegt bei |d| *=* 0,78 und entspricht damit einem mittleren Effekt.

Des Weiteren wurde gefragt, wie sehr sich die Studienteilnehmer*innen im Umgang mit Laptop/Notebook, Stand-PC, Tablet und iPad erfahren fühlen. Der Erfahrungswert sollte sich sowohl auf den beruflichen als auch auf den privaten Umgang beziehen. Die Auswertung zeigt, dass sich Psychotherapeut*innen bei t_1_ (völlig unerfahren 1: *N* *=* 14, 2: *N* *=* 8, 3: *N* *=* 52, 4: *N* *=* 59, äußerst erfahren 5: *N* *=* 42, *N*_gesamt_ *=* 175) und bei t_2_ (völlig unerfahren 1: *N* *=* 3, 2: *N* *=* 8, 3: *N* *=* 40, 4: *N* *=* 73, äußerst erfahren 5: *N* *=* 53, *N*_gesamt_ *=* 177) mit einem Median von 4 bei beiden Messzeitpunkten „erfahren“ fühlen. Betrachtet man die Messwerte aus der paarweisen Zuordnung, so zeigt sich, dass es keinen statistisch signifikanten Unterschied in der Erfahrung im Kontakt über Sprachtelefonie in Abhängigkeit des Messzeitpunkts gibt (asymptotischer Wilcoxon-Test: z *=* −1,804, *p* *=* 0,071, *n* *=* 53).

Weniger Erfahrung zeigte sich im Umgang mit neueren technischen Medien: Skype, Zoom, Facetime, WhatsApp, Signal, TheraPsy Connect, Instahelp, Telegram, Threema, fair-meeting, Jitsi Meet. Die Deskriptivstatistik zeigt, dass sich Psychotherapeut*innen bei t_1_ (völlig unerfahren 1: *N* *=* 47, 2: *N* *=* 112, 3: *N* *=* 16, 4: *N* *=* 0, äußerst erfahren 5: *N* *=* 0, *N*_gesamt_ *=* 175) und tendenziell etwas mehr bei t_2_ (völlig unerfahren 1: *N* *=* 3, 23: *N* *=* 131, 3: *N* *=* 20, 4: *N* *=* 3, äußerst erfahren 5: *N* *=* 0, *N*_gesamt_ *=* 177) mit diesen speziellen Medien in Summe mit einem Median von 2 bei beiden Messzeitpunkten „unerfahren“ fühlen. Die erkennbare Steigerungstendenz zeigt bei Betrachtung der Messwerte aus der paarweisen Zuordnung einen statistisch signifikanten Unterschied (asymptotischer Wilcoxon-Test: z *=* −2,887, *p* *=* 0,004, *n* *=* 53). Die Erfahrung im Kontakt über Videotelefonie ist bei t_2_ somit signifikant höher als bei t_1_ (Median t_1_ und t_2_: 2; völlig unerfahren 1: *N* zu t_1_ *=* 14, *N* zu t_2_ *=* 7; 2: *N* zu t_1_ *=* 36, *N* zu t_2_ *=* 40; 3 : *N* zu t_1_ *=* 3, *N* zu t_2_ *=* 6; 4: *N* zu t_1_ und t_2_ *=* 0; äußerst erfahren 5: *N* zu t_1_ und t_2_ *=* 0; *N*_gesamt_ *=* 53). Die Größe des statistischen Effekts liegt bei |d| *=* 0,78 und entspricht damit einem mittleren Effekt.

## Veränderung der therapeutischen Beziehung

Auf die Frage, welche Veränderungen Psychotherapeut*innen durch den Einsatz von TEP wahrnehmen, antwortet ein Großteil der Befragten beim ersten und zweiten Testzeitpunkt, dass sie die Qualität der therapeutischen Beziehung als verändert erleben (t_1_: 90 Angaben^3^, t_2_: 93 Angaben).

Als Qualitätsmerkmale der therapeutischen Beziehung werden in diesem Zusammenhang vorrangig Eigenschaften wie Intensität und die Möglichkeit zur persönlichen Öffnung, aber auch das Erleben von Distanz und einer gewissen Oberflächlichkeit genannt. Die Ergebnisse sind demnach sowohl in Richtung einer Intensivierung der Beziehung mit größerer persönlicher Offenheit zu sehen, als auch in Richtung einer erhöhten Distanzierung und Oberflächlichkeit der therapeutischen Beziehung.

So sprechen 24 (t_1_) bzw. 26 (t_2_) Therapeut*innen in diesem Zusammenhang von einer *Intensivierung der therapeutischen Beziehung*. Beispielsweise meint eine Gestalttherapeutin: „Die therapeutische Beziehung wird manchmal ‚privater‘, der therapeutische Setting-Rahmen [wird] als intimer wahrgenommen.“ (2099^4^, w^5^, IG^6^) Einzelne Befragte (t_1_: 4, t_2_: 9) geben zudem an, dass den Patient*innen durch den Einsatz von TEP die persönliche Öffnung leichter fällt. Etwas seltener wird die therapeutische Beziehung von den befragten Psychotherapeut*innen als distanzierter beschrieben (t_1_: 16, t_2_: 14), manchmal mit einer differenzierten Begründung, teilweise aber auch ohne. Diese Distanz tritt in manchen Fällen eher zu Beginn der virtuellen Sitzungen auf, was für einen Gewöhnungseffekt und eine Adaptierungsfähigkeit spricht. Es zeigt sich, dass die Distanz mit zunehmender Vertrautheit mit dem neuen Setting wieder abnimmt. Manche Befragten sprechen von mehr Oberflächlichkeit in der therapeutischen Beziehung und einer eingeschränkten Möglichkeit, diese zu vertiefen (t_1_:12, t_2_: 9).

Zu Beginn der Pandemie erleben doppelt so viele Psychotherapeut*innen Einschränkungen in der therapeutischen Beziehung wie beim zweiten Erhebungszeitpunkt (t_1_:80, t_2_: 41). Hier fällt die eingeschränkte Wahrnehmung der Sinneseindrücke hinein, welche sich durch den Einsatz von TEP ergibt (t_1_:22, t_2_: 9). Psychotherapie via „Telefon gestaltet sich schwierig, da alle wichtigen nonverbalen Signale fehlen und es leichter zu Missverständnissen kommt. Der persönliche Kontakt ist für mich nicht gleichwertig durch andere Medien zu ersetzen“ (540, w, PD). „Atmosphärisch ist weniger spürbar. Die zwischenleibliche Resonanz fehlt bzw. ist nur visuell oder akustisch wahrnehmbar“ (967, w, IG). Insgesamt findet „weniger Wahrnehmung von Körperausdruck, Stimmungen und Atmosphären“ statt (2655, w, IG).

## Behandlungstechniken

Um die Antworten auf die offenen Fragen der qualitativen Erhebung einzuordnen, wurden die Kategorien des generellen Selbsterlebens und in einer Spezifizierung die Unterkategorien des sprachlichen Erlebens, des szenischen Erlebens und des Körpererlebens gebildet, um einerseits der Heterogenität der psychotherapeutischen Verfahren der Stichprobe gerecht zu werden und andererseits allgemeine Tendenzen diesseits der Verfahrensunterschiede herauszuarbeiten.

Die Therapeut*innen geben am häufigsten an, dass sich bei TEP jene Techniken besonders gut eignen, die sich auf das sprachliche Erleben beziehen (t_1_:139, t_2_: 128). Dazu zählt, Begriffe assoziativ zu finden, als persönlich bedeutsam zu erfassen und verstehend einzuordnen und auch sprachlich adäquates und differenziertes Erleben im inneren Dialog respektive mit anderen Personen situationsgerecht zu nutzen. Am häufigsten wird hier der Begriff des Gesprächs genannt (t_1_:45, t_2_: 59). Des Weiteren nennen die Befragten oft das Zuhören (t_1_:16, t_2_: 10), Containing (t_1_:14, t_2_: 10) und Fragetechniken (t_1_: 12, t_2_: 8). Fragetechniken werden eher unspezifisch angeführt, zwei Psychotherapeut*innen bezeichnen es als „zirkuläres Fragen“ (905, m, SF; 2225, w, SF), ein Psychotherapeut spricht von „systemischen Fragetechniken“ (2873, m, NLPt). Darüber hinaus geben die Studienteilnehmer*innen an, in der TEP Psychoeduktion einzusetzen (t_1_:7, t_2_: 6) und strukturierend zu arbeiten (t_1_:6, t_2_: 4). Weitere Techniken sind: Reframing (t_1_:5, t_2_: 7), Schreiben (t_1_:4, t_2_: 4) wie etwa das „Schreiben von Tagebuch und Listen“ (360, w, IT), Sharing (t_1_:7, t_2_: 1), Zielfokussierung (t_1_:3, t_2_: 4), Reflexion (t_1_:3, t_2_: 4), Externalisieren (t_1_:2, t_2_: 2), Beziehungsarbeit (t_1_: 3, t_2_: 0).

Am zweithäufigsten nennen die Studienteilnehmer*innen, dass sich Techniken besonders gut eignen, welche sich auf das szenische Erleben beziehen (t_1_: 88, t_2_: 84). Szenisches Erleben meint das in der therapeutischen Situation aktiv imaginierte Entwickeln und Erfahren von Sequenzen mit sich und wichtigen anderen sowie die Aktivierung lebensgeschichtlicher Sequenzen als „Szenen“ der Erinnerung. Am häufigsten wird hier die psychotherapeutische Arbeit mit Imaginationen genannt (t_1_: 22, t_2_: 29). Die Psychotherapeut*innen sprechen in diesem Zusammenhang allgemein von Imaginationen, imaginativen Techniken oder Visualisierungen.

Techniken, die das Selbsterleben betreffen, werden am dritthäufigsten genannt (t_1_: 41, t_2_: 37). Das generelle Selbsterleben bezeichnet die ganzheitliche Selbsterfassung in ihrer leiblichen, psychischen und sozialen Situation und der speziellen, als Mentalisierungsfähigkeit bezeichneten Fähigkeit, mit sich und anderen gefühlshaft-selbstreflektierend verbunden und handelnd zu sein. Diesbezüglich sprechen sie von Techniken wie Ressourcenarbeit, die sie nicht genauer beschreiben (t_1_: 14, t_2_: 11) und der Arbeit mit Symbolen und Gegenständen (t_1_: 5, t_2_: 9). Darüber hinaus geben die Therapeut*innen an, dass sie Hausaufgaben einsetzen (t_1_: 8, t_2_: 4) und mit Skills (t_1_: 2, t_2_: 3), die Stressbewältigung und Spannungsregulation bewirken.

Am seltensten werden zunächst Techniken eingesetzt, die sich auf das Körpererleben der Patient*innen beziehen, wobei der Wert zum zweiten Messzeitpunkt deutlich zunimmt (t_1_: 27, t_2_: 43). Unter Körpererleben wird hier die aktuelle subjektive Wahrnehmung des Körpers im Kontakt mit sich selbst und in Bezug zu wichtigen anderen Personen verstanden, wobei subjektive Wahrnehmung die aktuellen Sinnesempfindungen als auch damit verbundene lebensgeschichtliche Körpererfahrungen meint, die in ihren unterschiedlichen Qualitäten situativ reaktiviert in das aktuelle Erleben mit einfließen und damit wesentlich die persönliche, subjektive Bedeutungsgebung der momentanen Situation beeinflussen. In diesem Zusammenhang führen Psychotherapeut*innen am häufigsten die Leibarbeit oder auch Körperarbeit an (t_1_: 6, t_2_: 10). Weitere Techniken sind Entspannungsübungen (t_1_: 6, t_2_: 8) oder Körperwahrnehmungsübungen (t_1_: 6, t_2_: 7).

## Diskussion und Ausblick

Die Auswahl der Psychotherapeut*innen dieser Studie könnte eine potenzielle Quelle für Verzerrungen sein, da keine repräsentative Stichprobe erhoben wurde. Der Link zum Fragebogen wurde nur an Psychotherapeut*innen des Österreichischen Arbeitskreises für Gruppentherapie und Gruppendynamik (ÖAGG) über E‑Mail geschickt. Dies ist insofern kritisch anzumerken, da so nur bestimmte psychotherapeutische Verfahren untersucht wurden und Fragen zur therapeutischen Beziehung sowie zu den Erfahrungen mit neuen Medien in der Psychotherapie und zu den Einschätzungen der Veränderungen der Themen in der Behandlung durch diese speziellen Verfahren beeinflusst sein könnten. So könnten zum Beispiel Veränderungen der therapeutischen Beziehung durch verhaltenstherapeutisch orientierte Psychotherapeut*innen, welche in dieser Umfrage nicht erfasst wurden, anders wahrgenommen bzw. erlebt werden. Die Erhebung mittels Online-Fragebogen bewirkt außerdem bis zu einem gewissen Grad, dass tendenziell Personen teilnehmen, die der elektronischen Datenverarbeitung und der Nutzung von Online-Tools aufgeschlossen begegnen. Damit verstärkt sich möglicherweise ein Problem, das auch in dem Review von Markowitz et al. (2020) benannt wird, nämlich dass bei Studien zu TEP vorwiegend Personen teilnehmen, die keine Berührungsängste mit Telekommunikation haben und damit dieser Behandlungsform eher wohlwollend begegnen. Entsprechend liegen dann vermehrt positive Rückmeldungen vor. Eine weitere, situationsbedingte Einschränkung bezüglich der Analyse der Ergebnisse ist, dass keine Daten zur Situation vor der COVID-19 Situation mit der gegenständlichen Stichprobe vorliegen.

Mit Blick auf die Umstellung zu TEP zeigt sich, dass die überwiegende Mehrheit der Befragten mit Einsetzen der COVID-19 Krise auf TEP umgestellt hat. Bemerkenswert ist, dass auch zum Zeitpunkt der Lockerung des Lockdowns zum Messzeitpunkt t_2_ eine Mehrheit weiterhin TEP oder gemischte Psychotherapie anwendet. Ob aus Sorge um die Gesundheit oder aufgrund der Wahrnehmung, dass TEP ein probates Mittel zur psychotherapeutischen Arbeit ist, müssen weitere Untersuchungen klären. Es zeigt sich, dass die Erfahrung über spezielle Apps und Videotelefonie signifikant zunimmt sowie Tendenzen bestehen, dass die Erfahrung mit neuen Medien im Allgemeinen zunimmt.

Zum ersten Messzeitpunkt t_1_ nach Beginn des Lockdowns werden 28 Angaben gemacht, die eine positive (Intensivierung und persönliche Öffnung) Veränderung der Qualität der therapeutischen Beziehung bekunden. In weiteren 28 Angaben wird von einer negativen (mehr Distanz und Oberflächlichkeit) Veränderung der Qualität der therapeutischen Beziehung berichtet. Zum zweiten Messzeitpunkt t_2_ werden 35 positive und 23 negative Angaben gemacht. Das bedeutet, dass entgegen der zugrunde gelegten Hypothese keineswegs eine allgemeine Tendenz zur Verschlechterung der Qualität der therapeutischen Beziehung von den Teilnehmenden wahrgenommen wird. Überraschend ist, dass sich mit der Zeit die Qualität der therapeutischen Beziehung laut den Angaben der Befragten im Rahmen von TEP auch verbessert. Auffällig ist, dass zu t_1_ 22 jedoch zu t_2_ lediglich 9 Psychotherapeut*innen von Einschränkungen bezüglich der vollsinnlichen Wahrnehmung sprechen. Dies spricht sicherlich für die Adaptierungsfähigkeit von Psychotherapeut*innen.

Allen Modifikationen der psychotherapeutischen Technik zum Trotz gilt bis zu einem gewissen Grad weiterhin, dass die Psychotherapie wesentlich eine *talking cure* ist und auf Versprachlichung zielt; auch in der Face-to-Face Situation. Es mag daher wenig verwundern, dass bezüglich der besonders geeigneten Techniken auch in der TEP am häufigsten das sprachliche Erleben genannt wird, gefolgt vom szenischen Erleben und Selbsterleben, wobei die Anzahl der Angaben in diesen drei Kategorien über die Messzeitpunkte eher konstant bleibt. Auffällig ist jedoch, dass die Anzahl der verwendeten Techniken in der Kategorie des Körpererlebens vom ersten zum zweiten Messzeitpunkt deutlich zunimmt und damit noch häufiger genannt wird, als Angaben zum Selbsterleben (t_1_: 27, t_2_: 43). Dies spricht mit Blick auf die Stichprobe unter den genannten Einschränkungen für eine hohe Adaptierungsfähigkeit von Psychotherapeut*innen und dafür, dass TEP, sowohl was die therapeutische Beziehung als auch Behandlungstechniken betrifft, eine wichtige Ergänzung zu Face-to-face Psychotherapie sein könnte. Die Ergebnisse der gegenständlichen Untersuchung legen damit nahe, in Ausbildung, Forschung und Gesetzgebung die TEP in Zukunft stärker zu berücksichtigen.

## Footnotes

1 Der Fragebogen umfasst insgesamt eine Länge von 25 Seiten in DIN A4 Format, weshalb dieser nicht im Anhang beigefügt wird. Auf Nachfrage kann dieser bei den Studienautor*innen angefragt werden.

2 Erhebung der Lebensqualität (WHOQOL-BREF, Angermeyer et al. 2000), der Resilienz (CD-RISC-10, Sarubin et al. 2015) und der Affektivität (PANAS, Janke und Glöckner-Rist 2012).

3 Die Anzahl hinter den Testzeitpunkten t_1_ und t_2_ beziehen sich hier auf verschiedene Wortmeldungen und nicht auf die Anzahl der Therapeut*innen.

4 Die Zahlen in Klammern beziehen sich auf den Fragebogen-Code, welcher in fortlaufenden Nummern angegeben wird.

5 „m“ bzw. „w“ geben das Geschlecht der Therapeutin an.

6 Nachfolgende Abkürzungen geben jenes psychotherapeutisches Verfahren an, welchem der*die Therapeut*in angehört. „nb“: nicht bekannt; „EL“: Existenzanalyse und Logotherapie; „IG“: Integrative Gestalttherapie; „IT“: Integrative Therapie; „KBT“: Konzentrative Bewegungstherapie; „PD“: Psychodrama; „DG“: Dynamische Gruppenpsychotherapie; „PA“: Psychoanalyse; „SF“: Systemische Familientherapie; „HY“: Hypnosepsychotherapie; „KIP“: Katathym Imaginative Psychotherapie; „KP“: Klientenzentrierte Psychotherapie; „PoP“: Psychoanalytisch orientierte Psychotherapie; „NLPt“: Neuro-Linguistische Psychotherapie; „GTP“: Gestalttheoretische Psychotherapie.
